# Invasive Group A Streptococcal Meningitis Following COVID-19 Infection Complicated by Multisystem Inflammatory Syndrome: A Case Report

**DOI:** 10.7759/cureus.71945

**Published:** 2024-10-20

**Authors:** Saya Hiramine, Yudai Tanaka, Chiaki Sano, Ryuichi Ohta

**Affiliations:** 1 Family Medicine, International University of Health and Welfare, Graduate School of Health Welfare Sciences, Tokyo, JPN; 2 Community Care, Unnan City Hospital, Unnan, JPN; 3 Community Medicine Management, Shimane University Faculty of Medicine, Izumo, JPN

**Keywords:** bacterial meningitis, covid-19, family medicine, general medicine, immunosuppression, multisystem inflammatory syndrome, older, parapharyngeal abscess, rural, streptococcus pyogenes

## Abstract

We present a case of a 60-year-old woman who developed invasive group A streptococcal (GAS) meningitis following a COVID-19 infection, complicated by multisystem inflammatory syndrome (MIS). Initially diagnosed with COVID-19 based on fever, nasal obstruction, cough, and sore throat, her symptoms improved with symptomatic treatment, except for a persistent sore throat. She later presented with hematemesis and was found to have bacterial pneumonia and dehydration. Despite treatment with ceftriaxone, her condition worsened with the development of a headache, shivering, and worsening respiratory and circulatory symptoms. Cerebrospinal fluid analysis confirmed bacterial meningitis, and treatment was escalated to include ceftriaxone, ampicillin, and vancomycin. An MRI revealed a parapharyngeal space abscess, and subsequent blood cultures identified GAS as the causative organism. The patient was treated with ampicillin for 14 days, followed by oral amoxicillin. Her condition improved, and she was discharged with no neurological deficits. This case underscores the need for vigilance in detecting secondary bacterial infections in post-COVID-19 patients, especially in MIS, where atypical presentations can delay diagnosis. Early recognition and aggressive treatment are vital to preventing complications and ensuring favorable outcomes.

## Introduction

Group A Streptococcus (GAS) is a Gram-positive bacterium commonly associated with upper respiratory tract infections and pyogenic skin conditions, typically colonizing the pharynx and skin of healthy individuals [1]. Infections usually arise from invasion through the pharynx and tonsils, with most cases being mild and resolving with appropriate oral antibiotics and follow-up care [2]. However, in specific populations, such as children, the elderly, and immunocompromised individuals, the infection can progress to severe invasive group A streptococcal disease [3]. These invasive infections can manifest in various anatomical locations, and detecting GAS in sites other than the pharynx is crucial for diagnosis.

Streptococcal meningitis, particularly caused by GAS, is an exceedingly rare form of bacterial meningitis, comprising only 1-2% of all cases. This contrasts with common causes, such as Streptococcus pneumoniae and Neisseria meningitidis, which are responsible for most bacterial meningitis cases globally. The rarity of GAS in meningitis makes it an exceptional clinical presentation, often linked to severe outcomes. A study from Denmark analyzing a large cohort of adult cases found that GAS meningitis carries a notably high mortality rate of 27% [4,5]. Despite its rarity, penicillin remains the first-line treatment.

In this report, we present a case of multisystem inflammatory syndrome (MIS) following COVID-19, complicated by invasive GAS infection. The diagnosis was challenging due to the atypical presentation: the patient developed persistent fever and headache following a COVID-19 infection but lacked the typical upper respiratory or skin manifestations of GAS, as well as classic meningitis symptoms such as altered consciousness or focal neurological deficits. We hypothesize that a transient immunocompromised state post-COVID-19 infection may have contributed to the disease progression. This case highlights the need for vigilance in recognizing and managing MIS and secondary bacterial infections in the post-COVID-19 setting.

## Case presentation

A 60-year-old woman presented to a rural community hospital with hematemesis as her chief complaint. Her symptoms began eight days prior with fever, nasal obstruction, cough, and sore throat. She was diagnosed with COVID-19 by her primary care physician and treated symptomatically with 500 mg of acetaminophen. Over time, her fever, nasal discharge, and cough resolved, but the sore throat persisted and was accompanied by nocturnal chills. Three days before admission, she experienced a single episode of vomiting. A primary care otolaryngologist had previously diagnosed her with viral pharyngitis and did not prescribe additional treatment. On review of her symptoms, there was no history of contact with sick individuals, travel abroad, unexplained weight loss, or night sweats. Her medical history included a gastric ulcer, cholelithiasis, and Helicobacter pylori infection, but she was not on any regular medications.

**Figure 1:**
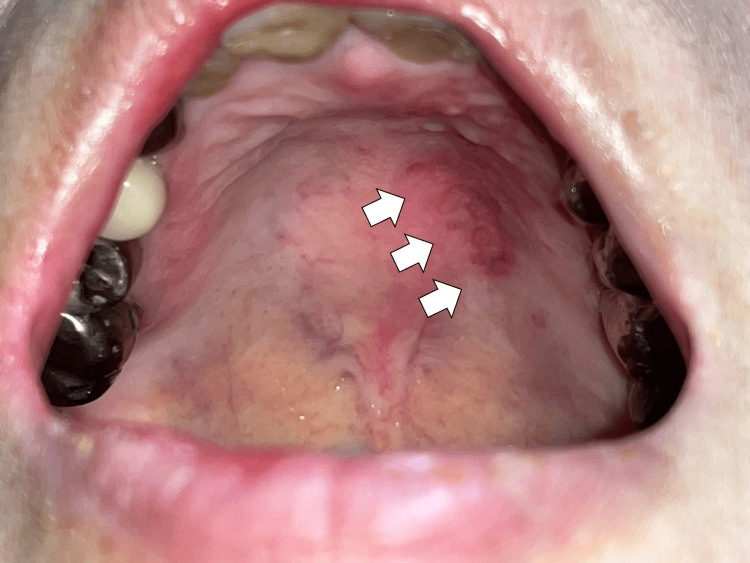
Oral ulcers on the hard palate (white arrows).

Upon arrival, her vital signs were as follows: temperature 36.7°C, blood pressure 111/70 mmHg, pulse 112 beats/min, respiration 16 breaths/min, and SpO2 97% on room air. She was alert with clear consciousness. Physical examination showed no cervical lymphadenopathy, positive jolt accentuation, and neck stiffness findings. Still, oral ulcers on the hard palate and late crackles on the right lower lung field were present (Figure 1).

Neurological examination was unremarkable, with a Glasgow Coma Scale (GCS) score of 15 (E4V5M6), and no focal neurological deficits were observed. A chest X-ray and 12-lead electrocardiogram showed no abnormalities. Echocardiography revealed inferior vena cava (IVC) collapse, suggestive of dehydration. Laboratory tests showed elevated neutrophils, hemoconcentration, and reduced renal function (Table 1).

**Table 1:** Initial laboratory data of the patient.

Parameter	Level	Reference
WBCs	13.10	3.5-9.1 × 10^3^ /μL
Neutrophils	86.3	44.0-72.0
Lymphocytes	7.1	18.0-59.0
Hemoglobin	16.6	11.3-15.2 g/dL
Hematocrit	48.1	33.4-44.9% (in %)
Mean corpuscular volume	88.3	79.0-100.0 fl
Platelets	17.4	13.0-36.9 × 10^4^ /μL
Total protein	8.5	6.5-8.3 g/dL
Albumin	3.4	3.8-5.3 g/dL
Total bilirubin	0.8	0.2-1.2 mg/dL
Aspartate aminotransferase	39	8-38 IU/L
Alanine aminotransferase	31	4-43 IU/L
Lactate dehydrogenase	256	121-245 U/L
Blood urea nitrogen	41.7	8-20 mg/dL
Creatinine	1.43	0.40-1.10 mg/dL
Serum Na	131	135-150 mEq/L
Serum K	3.1	3.5-5.3 mEq/L
Serum Cl	89	98-110 mEq/L
C-reactive protein	12.4	0.30 mg/dL

Sputum Gram stain revealed Gram-positive diplococci, leukocyte phagocytosis, and Gram-negative bacilli. Urine Gram stain showed a small number of Gram-positive streptococci but no leukocytes. Contrast-enhanced abdominal CT scan showed no active gastrointestinal bleeding. A diagnosis of post-COVID-19 bacterial pneumonia and dehydration was made, and treatment with ceftriaxone 2 g every 24 hours and intravenous fluids was initiated.

**Figure 2:**
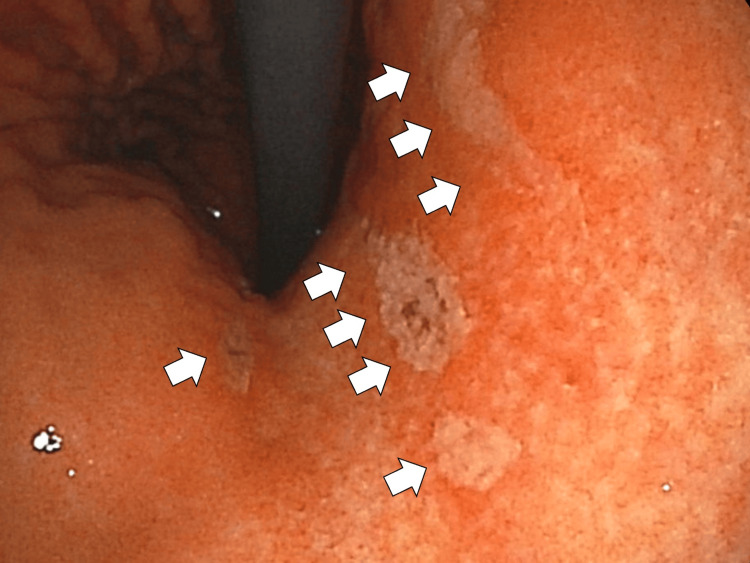
Upper gastrointestinal endoscopy revealing acute gastroduodenal mucosal lesions (white arrows).

On the second day of hospitalization, upper gastrointestinal endoscopy revealed nonsteroidal anti-inflammatory drug (NSAID)-induced acute gastroduodenal mucosal lesions (AGDML) in the healing phase (Figure 2).

Despite antimicrobial therapy, her fever persisted, and inflammatory markers worsened (C-reactive protein, 19.49 mg/dL; reference, <0.30). On the fourth day, tazobactam/piperacillin 4.5 g every 12 hours was added to cover possible enterococcal infection resistant to ceftriaxone. That evening, the patient developed shivering, worsening headache, and deteriorating respiratory and circulatory symptoms. A cerebrospinal fluid (CSF) test was performed, which revealed elevated cell counts, protein, and decreased glucose levels, consistent with bacterial meningitis (Table 2).

**Table 2:** The result of a cerebrospinal fluid analysis.

Parameter	Level	Reference
Opening pressure	17.5	10-25 cmH₂O
Cells	931	1-5/μL
Protein	63	15-45 mg/dL
Glucose levels	43	48-83 mg/dL

Considering the potential immunosuppressive state following COVID-19, she was treated with ceftriaxone 2 g every 12 hours for pneumococcal infection, ampicillin 2 g every 4 hours for Listeria and Enterococcus faecalis, and vancomycin 1 g/day for methicillin-resistant Staphylococcus aureus and multi-drug resistant gram-positive coccus.

**Figure 3:**
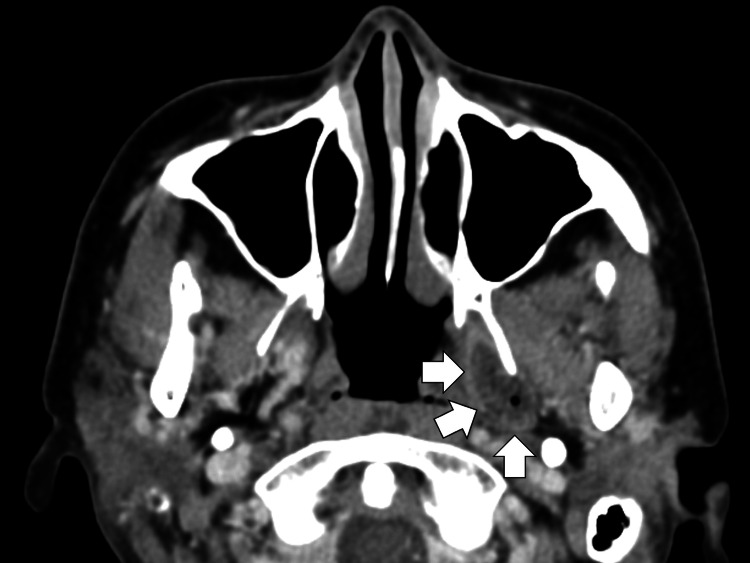
Contrast-enhanced head computed tomography showing a hypodense area with intralesional air and surrounding contrast-enhanced hyperdensity, indicative of a left parapharyngeal space abscess (white arrows).

On the fifth day, MIS was suspected, and corticosteroid therapy was initiated. A brain MRI was performed the same day to assess meningitis, and no central nerve lesions were observed. Contrast-enhanced head CT showed a hypodense area with intralesional air and surrounding contrast-enhanced hyperdensity, indicative of a left parapharyngeal space abscess (Figure 3).

**Figure 4:**
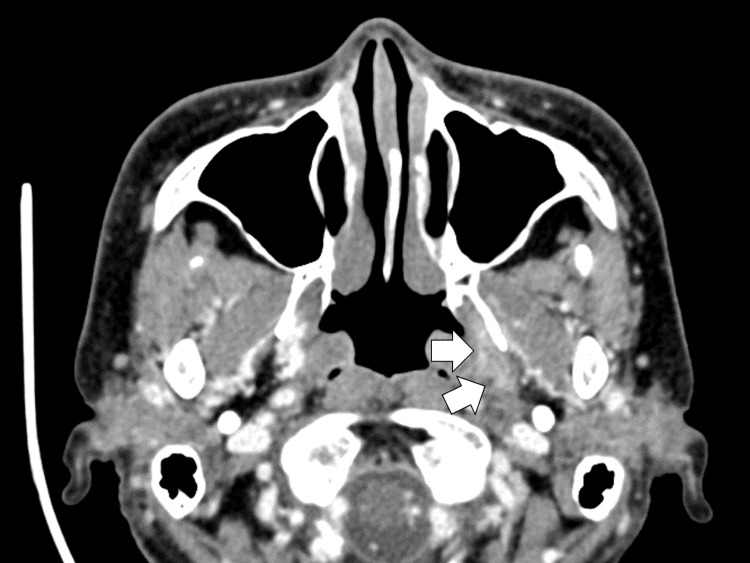
The contrast-enhanced head CT scan showed the shrinking of the left parapharyngeal abscess.

By the sixth day, her fever subsided, and her general condition improved with a trend toward recovery. On the seventh day, blood cultures grew GAS, and antibiotic therapy was de-escalated to ampicillin monotherapy of 6 g per day IV. She received ampicillin for 14 days, followed by oral amoxicillin 1500 mg per day. The patient's symptoms gradually improved, and her daily living activities were restored. She was discharged home on the 21st day of her illness with a continuation of oral antibiotic therapy. She was followed in the outpatient department on the 28th day, and the head CT scan with contrast showed the shrinking of the left parapharyngeal abscess (Figure 4).

Her treatment with oral antibiotics was completed on the 42nd day.

## Discussion

This case illustrates the potential for deep cervical infection to progress to meningitis following COVID-19, potentially exacerbated by complications of MIS, which can prolong immunosuppression and hyperinflammation [6]. If initial COVID-19 symptoms persist or if a hyperinflammatory state continues, the possibility of a secondary bacterial infection due to immunosuppression should be considered, and a thorough evaluation of the patient is warranted.

Effective management of COVID-19 requires vigilance for both the primary symptoms and the subsequent development of conditions like MIS and differentiation from long COVID. MIS-A, a subtype of MIS, involves an excessive immune response, potentially triggered by delayed viral clearance and prolonged interferon activation [7]. In contrast, long COVID is characterized by lingering symptoms such as fatigue, dyspnea, and brain fog, without the same intense systemic inflammation seen in MIS-A.

In our case, the patient exhibited persistent symptoms following a COVID-19 infection, with elevated inflammatory markers suggesting the possibility of MIS-A. Although more common in younger men, MIS-A has been reported in older adults, including those in their 70s [8]. It is distinct from long COVID due to its acute inflammatory presentation, with cardiovascular involvement (81%) being the most common, followed by the GI (73.4%) and skin/mucosal systems (51.9%) [9,10,11]. The prevalence of MIS-A ranges from 0.2% to 11.7%, depending on the study [9,10]. In this case, the patient's symptoms, including hematemesis, may have resulted from a combination of chronic gastritis due to H. pylori infection and mucosal damage related to MIS-A. Differentiating MIS-A from long COVID is critical, as MIS-A requires aggressive treatment for inflammation, while long COVID management is more symptom-focused. General physicians should remain alert to these differences when managing comorbidities in patients with prolonged post-COVID symptoms [12,13].

Secondary bacterial infections can occur due to a transiently weakened immune system following COVID-19, and close monitoring is necessary for patients with prolonged symptoms. In our case, Gram-positive diplococci were detected in the patient's sputum upon admission, suggesting bacterial pneumonia masked by dehydration, so ceftriaxone was initiated. Eventually, oral bacteria, including GAS, could enter the bloodstream through an impaired mucosal barrier caused by COVID-19-associated oral inflammation. Based on a previous article, the transient immunosuppression following COVID-19 can trigger invasive infections by normal flora [14]. In this case, the immunosuppression could have been severe, leading to the development of GAS meningitis. Despite positive blood cultures for GAS, the CSF culture did not show any growth, which may indicate that prior ceftriaxone therapy eradicated the bacteria in the CSF or that the inflammation of the CSF was triggered by MIS or inflammatory spread from a GAS abscess in the parapharyngeal space.

The diagnosis of GAS meningitis in this patient was challenging due to the atypical presentation and relatively mild disease severity. The rapid resolution of symptoms was likely attributable to the administration of appropriate antimicrobial therapy and the suppression of the inflammatory response with corticosteroids. In cases of prolonged fever and inflammatory response post-COVID-19, complicated by bacterial infection and organ damage, MIS should be suspected, and corticosteroid therapy should be considered despite its low prevalence [15]. Bacterial meningitis is a medical emergency requiring prompt diagnosis and treatment, as delays are directly associated with poor outcomes. It is estimated that only 44% of adults present with the four classic signs of bacterial meningitis (headache, fever, neck stiffness, and altered consciousness), and fewer than two-thirds exhibit the classic triad (fever, neck stiffness, and altered consciousness) [16,17]. Therefore, unconsciousness after COVID-19 should be investigated through cerebrospinal fluid tests, even if the physical examinations do not show signs of meningitis.

## Conclusions

This case highlights the complexity of managing post-COVID-19 complications, particularly in MIS and secondary bacterial infections. It underscores the importance of remaining vigilant for atypical presentations and recognizing the potential for severe infections such as GAS meningitis, even in the absence of classic symptoms. Early identification and prompt initiation of appropriate antimicrobial therapy, combined with supportive care, including corticosteroids for MIS, were crucial for achieving a favorable outcome in this patient. Clinicians should be aware of the immunosuppressive and hyperinflammatory states that can follow COVID-19, which may predispose patients to invasive bacterial infections. A multidisciplinary approach and thorough investigation are essential to improving prognosis in complex cases.
